# Diverse Response Pattern to Anoxia in Three Freshwater Turtle Species

**DOI:** 10.3390/biology12010050

**Published:** 2022-12-27

**Authors:** Min Li, Cuijuan Niu, Yixuan Chen

**Affiliations:** Ministry of Education Key Laboratory for Biodiversity Science and Ecological Engineering, College of Life Sciences, Normal University, Beijing 100875, China

**Keywords:** anoxia stress, antioxidant defense system, *Chinemys reevesii*, *Chelydra serpentina*, *Pelodiscus sinensis*

## Abstract

**Simple Summary:**

Three freshwater turtle species, the three-keeled pond turtle *Chinemys reevesii*, the snapping turtle *Chelydra serpentina* and the soft-shelled turtle *Pelodiscus sinensis*, often experience extensive changes in tissue oxygen levels in the field. This study measured total antioxidant capacity (TAOC), malondialdehyde (MDA) level (an index for oxidative damage) and parameters of the antioxidant defense system in the brain, liver and kidney of three turtle species to compare their antioxidant defense patterns to anoxia stress. Our results demonstrated different changing patterns in response to anoxia stress of the three freshwater turtle species. *C. reevesii* and *P. sinensis* were highly dependent on vitamin C for oxidative defense, while high activities of structural antioxidant enzymes were found in the tissues of *C. serpentina*.

**Abstract:**

With increasing water eutrophication and global warming, anoxia and hypoxia are becoming more and more common in water environments. Most vertebrates have a limited tolerance to anoxia of only a few minutes, but some species, such as turtles, can survive for months being exposed to anoxia. Antioxidant defense systems may have a potential role in resisting anoxia stress in freshwater turtles. The three-keeled pond turtle *Chinemys reevesii*, the snapping turtle *Chelydra serpentina* and the soft-shelled turtle *Pelodiscus sinensis* are three popular aquaculture species and share similar habitats in China. While *C. reevesii* and *C. serpentina* are hard-shelled turtles with poor skin permeability, *P. sinensis* is soft-shelled turtle whose skin permeability is good. We examined the antioxidant defense responses in different tissues of the three turtle species under acute anoxia stress for 10 h and subsequently recovered for 24 h in order to reveal the response patterns of the antioxidant defense system of the three turtle species that differed in morphological structure and life history strategy. We found that the antioxidant response patterns to acute anoxia stress were tissue- and species-specific. The soft-shelled turtle was more sensitive to anoxia than the hard-shelled turtles. Under anoxia stress, the three species kept the activities of most antioxidant enzymes stable. *C. reevesii* and *P. sinensis* were highly dependent on vitamin C in antioxidant defense, while high activities of structural antioxidant enzymes were found in the tissues of *C. serpentina*. The above diverse patterns may be related with adaptive evolution of morphological structure and physiological functions of the three turtle species.

## 1. Introduction

Many vertebrates inevitably experience anoxia stress across their life history, especially aquatic animals. Lots of lung-breathing aquatic animals severely deplete the oxygen stored in their bodies while diving, and some aquatic ectotherms spend the winter under ice, where the dissolved oxygen (DO) is decreased substantially because of blocked oxygen supplement from the air by ice. In recent years, with the aggravation of water pollution and the impact of global warming, DO content in water has been further reduced [1], and the survival of aquatic animals has to face the increasing probability of exposure to anoxia. The characteristics of water anoxia generally refer to DO concentration lower than 2 mg/L [2,3].

Freshwater turtles, as lung-breathing diving animals, often experience extensive changes in tissue oxygen levels. Their organs are subjected to anoxia pressure during diving, especially during prolonged diving, where circulatory regulation may result in preferentially direct oxygenated blood to vital organs, leading to profound anoxia in many other organs [4,5]. Moreover, freshwater turtles in temperate zones usually hibernate underwater during the cold winter, which is characterized by long-term, severe anoxia. The impact of global warming and the aggravation of water pollution pose further challenges to the underwater respiration of turtles. However, many freshwater turtles are excellent facultative anaerobe that can survive for several weeks without oxygen, such as those reported by Ultsch (1985, 1989) [6,7] which survived at least 2–3 months in water with complete anoxia at 3 °C.

The Chinese three-keeled pond turtle *Chinemys reevesii*, the snapping turtle *Chelydra serpentina* and the Chinese soft-shelled turtle *Pelodiscus sinensis* are all freshwater turtle species. Among them, the pond turtle and the soft-shelled turtles are widely distributed native species in China, and the snapping turtle is imported from North America. They belong to different families but live in similar habitats, and they have great differences in morphological structure. Among them, *C. reevesii* and *C. serpentina* are hard-shelled turtles, while *P. sinensis* is a soft-shelled turtle. In North America, some soft-shelled turtles, such as spiny softshell turtle *Apalone spinifera*, are found only in large lakes and riverine systems which maintain normoxic conditions throughout the year, and this habitat restriction may be due to their inability to tolerate prolonged anoxic diving [8,9,10,11]. In contrast, the snapping turtle is found in most permanent bodies of water, including eutrophic waters, which become hypoxic or anoxic in winter [12]. However, studies concerning how freshwater turtles’ antioxidant defense system respond to anoxia stress are still scarce. Alibardi and Toni (2006) [13] found that wild snapping turtles in North America coped well with the stress caused by an anoxic environment and lived for more than 100 days under low temperature and in an anoxic environment. Zhan et al. (2010) [14] reported that Chinese three-keeled pond turtles survived for 30 h in 7 L of anoxic water at 30 °C. There has been no report on anoxic tolerance of Chinese soft-shelled turtles.

Numerous studies have demonstrated that freshwater turtles have strong tolerance to external stresses, which may benefit from their highly effective antioxidant defense system to clear excess reactive oxygen species (ROS) produced by stress [11,15]. During winter dormancy, the painted turtle *Chrysemys pictacan* can survive for 4 months without oxygen. The mRNA levels of glutathione peroxidase 1, glutathione peroxidase 4 and glutathione reductase 1 in the heart and liver of *C. picta marginata* were significantly increased in response to anoxia stress [16]. This result proves that the antioxidant defense system of freshwater turtles is vital to withstand anoxia. However, studies on how the antioxidant defense system responds to anoxia in subtropical-originated turtles are still insufficient, especially comparative studies.

We measured total antioxidant capacity (TAOC) and malondialdehyde (MDA) level (an index for oxidative damage) in the brain, liver and kidney; the antioxidant enzyme gene expressions (superoxide dismutase SOD, catalase CAT and glutathione peroxidase GPx) in the brain; the antioxidant enzyme activities in the brain, liver and kidney; and the content of small-molecule antioxidants (glutathione: GSH, vitamin C: VC) in the tissues and plasma of the above three turtle species to compare their antioxidant defense patterns to anoxia stress. We predicted that the soft-shelled turtle *P. sinensis* was the most sensitive one to anoxia stress and that the response patterns of antioxidant defense system in different turtles were species- and tissue-specific under anoxia stress.

## 2. Materials and Methods

This study was approved by the Animal Ethics Committee of the College of Life Sciences, Beijing Normal University, and is in line with the relevant regulations of the National Experimental Animal Welfare Ethics and the Management Measures for the Working Permit of Experimental Animal Practitioners in Beijing (Approval No. 1120111900020).

### 2.1. Experimental Animals

Turtles of the three turtle species were purchased from a turtle hatchery facility (Yangzhou, Jiangsu, China, and Shaihai, China). We ignored the effect of gender on the experiment because the turtle juveniles had no phenotypic sexual differences. Animals were reared in a recirculating water system, the photoperiod was held at 12 L/12 D and temperature was kept at 28 ± 1 °C. They were fed daily with commercial feed at 12:00 PM.

### 2.2. Anoxia Stress and Recovery Experiments

After 4 weeks of acclimation, 30 healthy turtles were selected for the experiment in each species (*C. reevesii*: 93.57 g ± 2.11 g; *C. serpentina*: 119.76 ± 1.83 g; *P. sinensis*: 81.48 ± 2.47 g). All turtles were fasted for 24 h before treatment and were fasted throughout the whole treatment. In this study, we designed three sets of experiments.

A preliminary experiment showed that the experimental animals began to die after 12 h of acute anoxia, so we set the anoxia stress time to 10 h. Firstly, we transferred the control group turtles to a transparent water tank with saturated DO for 10 h, respectively (*C. reevesii*, *n* = 10; *P. sinensis*, *n* = 10; *C. serpentina*, *n* = 10). Secondly, the anoxia group turtles were transferred to a transparent water tank with DO below 2 mg/L for 10 h. Thirdly, the recovery group also experienced anoxia stress for 10 h and then recovered in saturated DO water for 24 h.

Turtles were sampled soon after the treatment. The brain, liver and kidney tissues and plasma were collected, frozen in liquid nitrogen and transferred to a −80 °C freezer for later analysis; for details please see Chen et al. (2022) [17].

**Figure 1:**
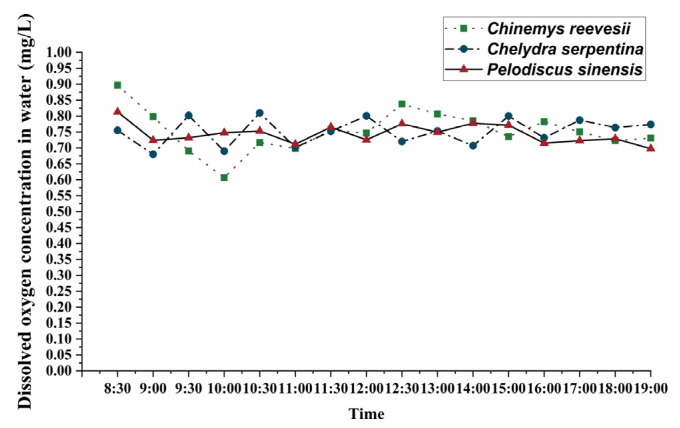
Diagram of dynamic monitoring of water oxygen content.

Dissolved oxygen levels in water during anoxia treatment were tested by an oxygen sensor (FireStingO_2_, Pyro Science, Aachen, Germany), and the results are shown in Figure 1.

### 2.3. Cerebral Antioxidant Enzyme Genes’ mRNA Levels

Cerebral mRNA levels of antioxidant enzyme genes, including Cu/Zn SOD (GenBank accession No. JX470524), Mn SOD (GenBank accession No. JX470525), CAT (GenBank accession No. JX452102), GPx1 (GenBank accession No. KC357250), GPx3 (GenBank accession No. JX470527) and GPx4 (GenBank accession No. JX470528) were measured by RT-qPCR analysis using 7500 real-time PCR system (Applied Biosystems, Carlsbad, CA, USA). GAPDH (GenBank accession No. NM_001286927.1) was used as an endogenous reference to normalize the template amount. The gene-specific primers used for mRNA quantification by RT-qPCR are shown in Table 1. The RNA extracting, cDNA synthesis and RT-qPCR processes were carried out as Chen et al. (2015) [15].

### 2.4. Biochemical Assays

Tissues were homogenized in phosphate-buffered solution on ice. Then, the homogenates were centrifuged and the supernatants were collected for biochemical analysis. Diagnostic Reagent Kits (Nanjing Jiancheng, Nanjing, Jiangsu, China) were used to measure the content of MDA, TAOC levels, enzyme activities (SOD, CAT and GPx) and the content of small-molecular antioxidants (GSH and VC) according to the instruction manual for each kit.

Fe^3+^ can be reduced to Fe^2+^ by antioxidants in the tissues; the latter can form a stable complex with phenanthrolines, and we can measure the level of TAOC in the absorption value at 520 nm [18]. MDA was measured based on the reaction of MDA and 2-thiobarbituric acid in which a product with an absorption peak at 532 nm could be determined [19]. The superoxide radicals generated in the xanthine–xanthine oxidase system can inhibit cytochrome c reduction, and we measured the activity of SOD in the absorption value at 550 nm [20]. The activity of CAT was measured by determining the level of H_2_O_2_ (μmol) decomposed in 1 g protein per second with changes of absorption value at 405 nm. GPx activity was determined by measuring the coupled oxidation of NADPH during glutathione reductase recycling of oxidized glutathione from GPx-mediated reduction of t-butyl peroxide, and one unit was defined as the amount of enzyme that oxidizes 1 μmol of NADPH per minute. Additionally, 5,5’-Dithiobis-(2-nitrobenzoic acid) (DTNB) can react with GSH to generate DTNB and GSSG. By measuring the absorption value at 405 nm, we calculated the concentration of GSH in tissues. Fe^3+^ can react quickly with reduced ascorbic acid to produce Fe^2+^, and then Fe^2+^ can react with phenanthroline. By measuring the absorption value at 536 nm, VC content in tissues and plasma was calculated. Protein concentration was measured according to Bradford (1976) [21], and bovine serum albumin was used as the standard. All enzyme assays were performed at 25 °C.

### 2.5. Statistical Analysis

Statistical analysis was conducted with SPSS 19.0. Data were presented as mean ± SE and 0.05 was set as statistical significance. Data were checked for normality and homogeneity of variance. If these assumptions were met, one-way analysis of variance (ANOVA) was used for comparative analysis, and Turkey’s HSD multiple-range post-hoc test was used when both assumptions were met. If the data showed unsatisfactory normality and homogeneity of variance, the Kruskal–Wallis test, followed by the Mann–Whitney U post-hoc test, were used. Using Origin 9.0 was used to plot data analysis results.

## 3. Results

### 3.1. TAOC Level

**Figure 2:**
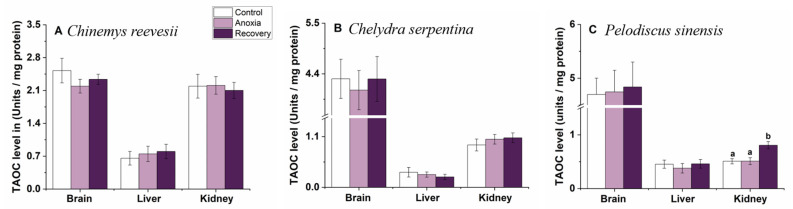
Effect of anoxia stress on TAOC level in tissues of *C. reevesii* (**A**), *C. serpentina* (**B**) and *P. sinensis* (**C**). Data are presented as mean ± SE. N = 10. Data with no common lowercase letters on the bar indicate significant differences between different groups (*p* < 0.05).

During the treatment, TAOC levels did not change in *C. reevesii* or *C. serpentina* (*p* > 0.05, Figure 2A,B). However, in *P. sinensis*, TAOC increased significantly in the kidney after recovery (one-way ANOVA, F_2,25_ = 7.743, *p* = 0.002) and did not change in the liver or brain (Figure 2C).

### 3.2. MDA Concentration

**Figure 3:**
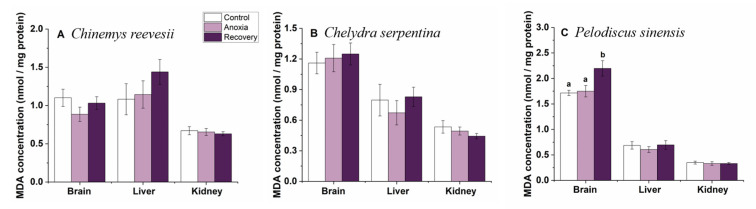
Effect of anoxia stress on MDA concentration in tissues of *C. reevesii* (**A**), *C. serpentina* (**B**) and *P. sinensis* (**C**). Data are presented as mean ± SE. N = 10. Data with no common lowercase letters on the bar indicate significant differences between different groups (*p* < 0.05).

During the treatment, MDA concentration did not change in *C. reevesii* or *C. serpentina* (*p* > 0.05, Figure 3A,B). However, in *P. sinensis*, cerebral MDA concentration increased significantly after recovery (one-way ANOVA, F_2,27_ = 5.720, *p* = 0.008), and did not change in the liver or kidney (Figure 3C).

### 3.3. Gene mRNA Expression of Cerebral Antioxidant Enzymes

**Figure 4:**
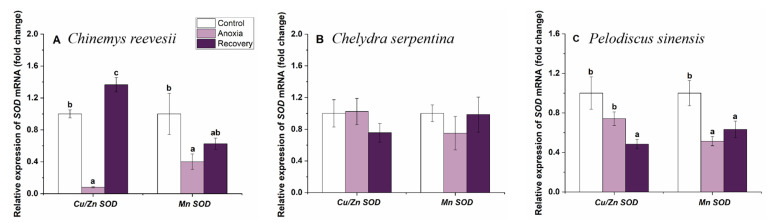
Changes of anoxia stress on related expression of SOD mRNA in the brain of *C. reevesii* (**A**), *C. serpentina* (**B**) and *P. sinensis* (**C**). Data are presented as mean ± SE. N = 8. Data with no common lowercase letters on the bar indicate significant differences between different groups (*p* < 0.05).

During anoxia, the expression of Cu/ZnSOD and MnSOD genes in the brain of *C. reevesii* decreased significantly. However, Cu/Zn SOD gene expression increased significantly after recovery, even higher than the control group (Cu/ZnSOD, F_2,19_ = 115.943, *p* < 0.001; MnSOD, χ2 = 7.042, df = 2, *p* = 0.030) (Figure 4A). In the whole experimental process, cerebral SOD mRNA levels did not change in *C. serpentina* (*p* > 0.05, Figure 4B). In *P. sinensis*, the expression of the Cu/Zn SOD gene decreased significantly after recovery (F_2,20_ = 5.893, *p* = 0.010), and the expression of the MnSOD gene decreased significantly during anoxia and remained at a low level after recovery (F_2,20_ = 7.147, *p* = 0.005) (Figure 4C).

**Figure 5:**
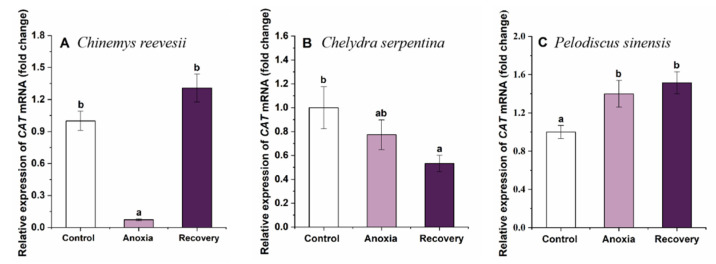
Changes of anoxia stress on related expression of CAT mRNA in the brain of *C. reevesii* (**A**), *C. serpentina* (**B**) and *P. sinensis* (**C**). Data are presented as mean ± SE. N = 8. Data with no common lowercase letters on the bar indicate significant differences between different groups (*p* < 0.05).

During anoxia, CAT gene expression lowered significantly in *C. reevesii* and recovered after recovery (F_2,20_ = 42.525, *p* < 0.001) (Figure 5A). The expression of CAT decreased significantly after recovery in *C. serpentina* (F_2,21_ = 3.216, *p* = 0.019) (Figure 5B), while in *P. sinensis*, CAT expression changed oppositely (F_2,20_ = 5.237, *p* = 0.015) (Figure 5C).

**Figure 6:**
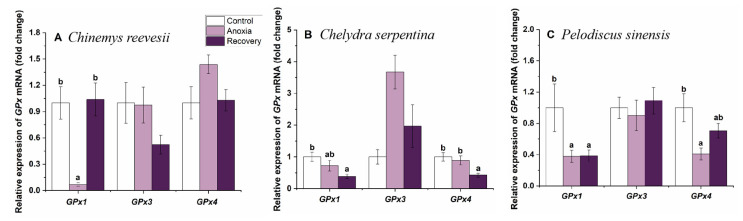
Changes of anoxia stress on related expression of GPx mRNA in the brain of *C. reevesii* (**A**), *C. serpentina* (**B**) and *P. sinensis* (**C**). Data are presented as mean ± SE. N = 8. Data with no common lowercase letters on the bar indicate significant differences between different groups (*p* < 0.05).

In *C. reevesii*, the expression of GPx1 decreased significantly during anoxia and recovered after recovery (F_2,21_ = 13.282, *p* < 0.001) (Figure 6A). In *C. serpentina*, the expression of GPx1 and GPx4 reduced significantly after recovery (GPx1, F_2,18_ = 5.353, *p* = 0.015; GPx4, F_2,21_ = 6.706, *p* = 0.006) (Figure 6B). In *P. sinensis*, the expression of GPx1 and GPx4 went down significantly during anoxia, and the expression of GPx4 recovered after recovery (GPx1, F_2,20_ = 4.163, *p* = 0.031; GPx4, F_2,21_ = 5.696, *p* = 0.011) (Figure 6C).

### 3.4. Antioxidant Enzymes

**Figure 7:**
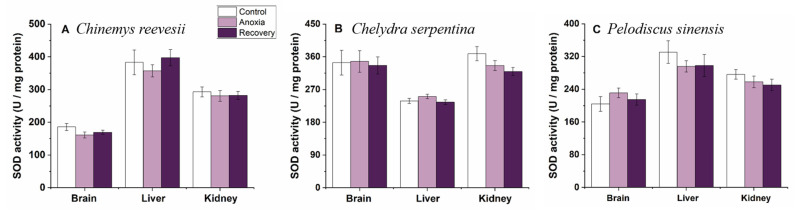
Changes of anoxia stress on SOD activity in tissues of *C. reevesii* (**A**), *C. serpentina* (**B**) and *P. sinensis* (**C**). Data are presented as mean ± SE. N = 10. Data with no common lowercase letters on the bar indicate significant differences between different groups (*p* < 0.05).

**Figure 8:**
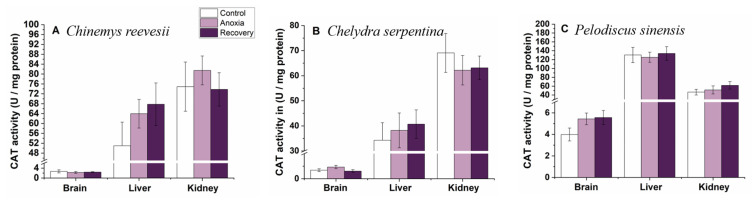
Changes of anoxia stress on CAT activity in tissues of *C. reevesii* (**A**), *C. serpentina* (**B**) and *P. sinensis* (**C**). Data are presented as mean ± SE. N = 10. Data with no common lowercase letters on the bar indicate significant differences between different groups (*p* < 0.05).

SOD and CAT activities did not change in any tissues of all three turtle species during the whole experimental process (*p* > 0.05, Figure 7 and Figure 8).

**Figure 9:**
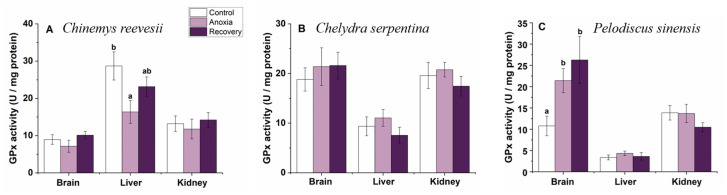
Changes of anoxia stress on GPx activity in tissues of *C. reevesii* (**A**), *C. serpentina* (**B**) and *P. sinensis* (**C**). Data are presented as mean ± SE. N = 10. Data with no common lowercase letters on the bar indicate significant difference between different groups (*p* < 0.05).

Hepatic GPx activities decreased significantly during anoxia then recovered after recovery (F_2,25_ = 3.837, *p* = 0.035) but remained stable in the brain and kidney in *C. reevesii* (*p* > 0.05, Figure 9A). During the treatment, GPx activities did not change in any tissues of *C. serpentina* (*p* > 0.05, Figure 9B). In *P. sinensis*, cerebral GPx activity increased clearly during anoxia and still kept the high level after recovery (F_2,22_ = 2.409, *p* = 0.040), but changed little in the liver and kidney during the whole treatment (*p* > 0.05, Figure 9C).

### 3.5. Small-Molecule Antioxidants

**Figure 10:**
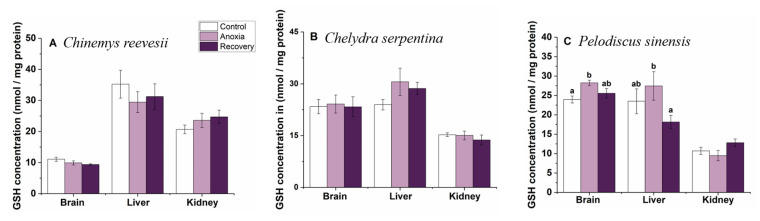
Changes of anoxia stress on GSH content in tissues of *C. reevesii* (**A**), *C. serpentina* (**B**) and *P. sinensis* (**C**). Data are presented as mean ± SE. N = 10. Data with no common lowercase letters on the bar indicate significant differences between different groups (*p* < 0.05).

GSH concentration did not change in all examined tissues of *C. reevesii* and *C. serpentina* during the whole treatment (*p* > 0.05, Figure 10A,B). In *P. sinensis*, cerebral GSH concentration increased significantly during anoxia and recovered after recovery (F_2,27_ = 5.150, *p* = 0.013), while hepatic GSH concentration slightly increased during anoxia then decreased significantly after recovery (F_2,27_ = 2.430, *p* = 0.107); no change was found in the kidney (*p* > 0.05, Figure 10C).

**Figure 11:**
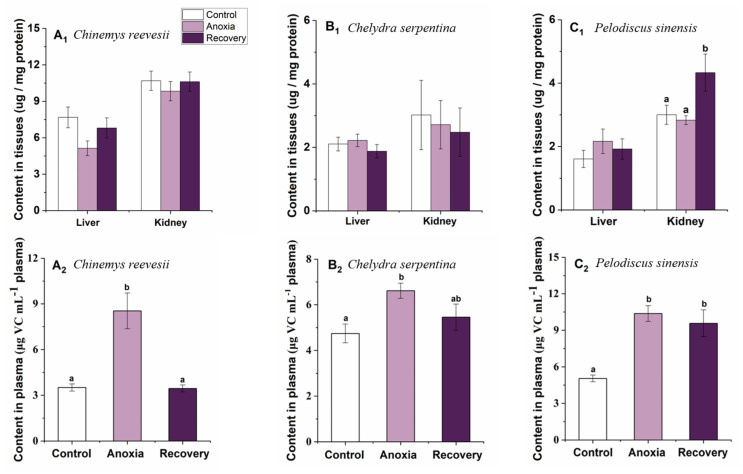
Changes of anoxia stress on VC content in tissues and plasma of *C. reevesii* (**A**), *C. serpentina* (**B**) and *P. sinensis* (**C**). Data are presented as mean ± SE. N = 10. Data with no common lowercase letters on the bar indicate significant differences between different groups (*p* < 0.05).

Anoxia stress did not affect VC content in the liver and kidney of *C. reevesii* and *C. serpentina* or in the liver of *P. sinensis* (*p* > 0.05, Figure 11). Vitamin C increased significantly in the kidney after recovery in *P. sinensis* (F_2,20_ = 3.604, *p* = 0.046) (Figure 11C1). Plasma VC contents of the three turtle species were significantly affected by anoxia, and all increased significantly during the anoxia period (*p* > 0.05). After recovery, plasma VCs in *C. reevesii* and *C. serpentina* recovered to the control level, while that of *P. sinensis* remained at the high level (F_2,28_ = 14.217, *P_C. Reevesii_* < 0.001; F_2,30_ = 4.126, *P_C. Serpentina_* = 0.027; F_2.25_ = 11.515, *P_P. Sinensis_* < 0.001) (Figure 11).

### 3.6. Comparison of the Response Pattern to Anoxia Stress among the Three Turtle Species

**Figure 12:**
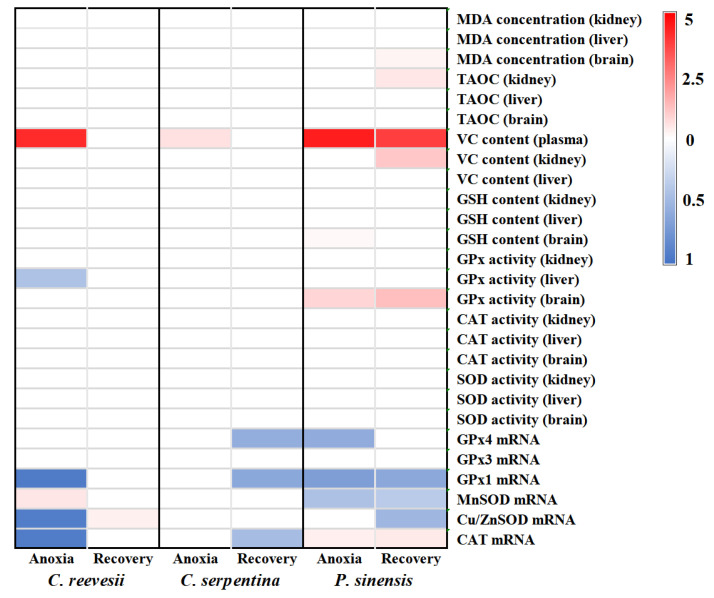
Sketch showing the changing pattern of different parameters in the three turtle species under anoxia stress. Note: Each row in the figure corresponds to an indicator, and each column corresponds to an experimental group. The variation range of data is 0–5. Only indicators with significant differences are marked in color. White: there is no significant difference between the treatment group and the control group. Red: compared with the control group, the treatment group increased significantly, and the darker the red, the greater the increase. Blue: compared with the control group, the treatment group decreased significantly, and the darker the blue, the greater the decrease.

To compare the differences in antioxidant response patterns to anoxia stress among the three turtle species, we examined changes of all parameters compared to the control level, and the results are shown in Figure 12.

Our results show that antioxidant responses of the turtles were tissue- and species-specific. Most of the antioxidant enzyme activities of the three turtle species did not change during anoxia exposure. However, the expression of antioxidant enzyme genes in the brain and the content of small-molecule antioxidants fluctuated significantly in response to anoxia stress. The Chinese soft-shelled turtle was the most sensitive species to anoxia stress among the three turtles, with increased cerebral MDA concentration and renal TAOC. The number of changed antioxidant parameters was also the most in the soft-shelled turtle among the three turtle species in the treatment.

## 4. Discussion

Compared with endothermic birds and mammals, most ectothermic vertebrates (fishes, amphibians and reptiles) can withstand a much wider range of changes in oxygen levels. Some fishes and turtles are extremely anoxia-tolerant vertebrates; they can survive in completely anoxic conditions for a long time. Perhaps the most famous example is the painted turtle (*Chrysemys picta*), a North American species that can survive without oxygen for up to 4 months during winter hibernation [6,22]. Animals facing anoxia have to deal with oxidative stress when oxygen re-enters their bodies, which will result in ROS surges after a long period of lack of oxygen [23,24,25,26,27,28,29,30,31,32,33]. In the process of restoring oxygen supply, animals may adopt different strategies to cope with ROS production. These strategies include: (1) maintaining a sustained high level of antioxidant defense and timely removal of the excessive ROS; (2) stress-induced adjustment of antioxidant defense system to cope with the oxidative stress that may occur after the resumption of oxygen; (3) tolerance to higher levels of free radical damage or enhanced mechanisms for scavenging damaged products [24].

Studies have shown that compared with hard-shelled turtles, soft-shelled turtles are more sensitive to anoxia [11]. In the present study, we found that when exposed to 10 h anoxia stress and then recovered 24 h, MDA content in the brain of *P. sinensis* increased significantly, resulting in oxidative damage, while *C. reevesii* and *C. serpentina* showed no MDA variations, indicating that *P. sinensis* was more sensitive to anoxia stress. Chen et al. (2021) found that after exposure to ammonia stress for 96 h, the blood ammonia level of *P. sinensis* was significantly higher than that of the other two turtle species, indicating that *P. sinensis* was more sensitive to environmental change [17]. It is speculated that this may be related to the leathery skin of *P. sinensis*. Under normal circumstances, *P. sinensis* can still use leathery skin to breathe even when submerged in water. Therefore, the probability of experiencing anoxia in its life history is lower than that of the others, and the body lacks more effective anoxia coping strategies.

ROS are mostly produced in the mitochondrial respiratory chain, and they are byproducts of aerobic metabolism. Animals are often at risk of outbreaks of ROS after prolonged periods of anoxia followed by the resumption of oxygen supply. The antioxidant defense system, including the antioxidant enzyme system and the small-molecule antioxidant system, plays an important role in stress tolerance and antioxidant homeostasis maintenance [25]. The brain is consumes the most oxygen per gram of tissue, and despite representing only 2% of the body weight, it uses 20% of the total oxygen consumption [26]. Therefore, maintaining the redox balance of the brain in a stable state is necessary for the body to maintain normal life activities. In this study, the expression of antioxidant enzyme genes in the brain of three turtle species showed a large fluctuation but varied in changing pattern among species. During anoxia, the expression of antioxidant enzyme genes in the brain of *C. reevesii* decreased significantly and then increased after recovery, which was in line with our prediction. Metabolism slowed down during anoxia, and the possibility of ROS generation decreased, so the expression of antioxidant enzyme genes decreased during anoxia. After the resumption of oxygen, the metabolism of the body gradually returned to normal levels, and with the risk of ROS outbreak, the expression of antioxidant enzyme genes increased significantly after recovery. These results were in line with the study of Zhang et al. (2022) [27], who found that the expression of Cu/Zn-SOD and Mn-SOD genes in the heart of silver carp (*Hypophthalmichthys molitrix*) was significantly lower than that of normoxia at 24 h of hypoxia stress and returned to normoxia levels after reoxygenation. In contrast, cerebral antioxidant genes’ expression of *C. serpentina* did not change during anoxia but decreased after recovery in the present study, perhaps due to the fact that because of higher levels of structural antioxidant enzyme activities in the brain (Figure 9B) and the higher hemoglobin level among the three turtle species [17], it is not necessary to spend extra energy on regulation of antioxidant enzymes after recovery, so the expression of antioxidant enzyme genes remained stable during the whole treatment. In *P. sinensis*, cerebral CAT mRNA expression significantly increased and remained at this high level after recovery. Similar results were found in shrimp (*Litopenaeus vannamei*); after exposure to hypoxia for 4 h, the expression of cMnSOD and GPx in the hepatopancreas increased significantly [28]. Such “anticipatory” increases in antioxidant gene expression during reoxygenation have been reported in various intertidal and estuarine organisms [29,30,31]. However, GPx1 and GPx4 genes’ expression clearly decreased during anoxia, while SOD mRNA levels reduced markedly after recovery. It seemed that CAT played the most important role in removing ROS, and the decreased expression of GPx1, GPx4 and SOD suggested a selective downregulation of some antioxidants’ transcripts and not a global decrease in metabolism [28].

Turtles have been considered to have a strong ability to withstand anoxia [32]. Willmore and Storey (1997) [33] found that after 20 h of anoxia, SOD activity in the liver and CAT activity in the kidney of freshwater turtle (*Trachemys scripta elegans*) decreased significantly and then recovered after recovery, which was consistent with the change of GPx activity in the liver of the pond turtles in our study. This may be because metabolism slows down during anoxia, which leads to the blocking of enzyme synthesis, and metabolism accelerates after oxygen is restored. In addition, the possibility of ROS injury is relatively lower under hypoxia. There were no significant changes in the activities of antioxidant enzymes (CAT, SOD and GPx) in the tissues of *C. serpentina* during the whole experiment, and *C. serpentina* has higher levels of structural antioxidant enzymes in tissues than the other two turtles; after anoxia stress, enzyme activity remained at high levels, which can remove excess ROS timely. These results corresponded to the living environment of snapping turtles; as diving animals, the oxygen tension of blood and organs in snapping turtles often faces a wide range of changes, and anoxia exposure on time and duration may be frequent and unpredictable. A possible strategy for adapting to this lifestyle is to maintain high levels of antioxidant defense ability to limit ROS production after frequent anoxia or resumption of oxygen supply. Anoxia stress conditions include hypoxia, anoxia, freezing, severe dehydration and exposure of water-breathing animals to air. Many studies have shown that when some animals are exposed to hypoxic stress, their antioxidant defenses are enhanced [31,34]. For example, Lushchak et al. (2001) [35] found that after anoxia treatment for 8 h, SOD activity in the brain of goldfish (*Carassius auratus*) increased significantly. After 5.5 h of anoxia, the activity of CAT and SeGPx in the brain of common carp (*Cyprinus carpio*) clearly increased [36]. In this study, we found that cerebral GPx activity increased significantly during anoxia and continued to rise after recovery to reduce oxidative injury. The reason for the inconsistency between GPx enzyme activity and gene expression in *P. sinensis* may be that gene expression is regulated by negative feedback of enzyme activity [37]. At this sampling point, ROS was effectively eliminated or the existing GPx activity was sufficient to remove excess ROS in the brain.

In the present study, we found that the gene expressions of antioxidant enzymes were not completely consistent with the activities in the brain. To be specific, during anoxia, the expression of antioxidant enzyme gene in the brain of *C. reevesii* decreased significantly and then increased after recovery, while most of its activities remained stable. Cerebral antioxidant gene expressions of *C. serpentina* did not change during anoxia and decreased after recovery in the present study, but the activities of the three antioxidant enzymes remained stable during the whole treatment. As for *P. sinensis*, the gene expressions of antioxidant enzymes fluctuated significantly, while the enzyme activities remained mostly stable. Inconsistency between enzyme gene expression and enzyme activity is a common phenomenon. The reasons for this situation may be as follows: (1) gene expression is transient [38], which is only reflected in the situation of the gene at the certain sampling point, while the following changes in enzyme activities are usually time-lagged; (2) gene expression is subject to negative feedback regulation by enzyme activity [22].

Studies have found that VC plays an important role in the antioxidant defense system of *P. sinensis* and *C. reevesii* [15,39]. Our results showed that among the measured tissues of the three turtles, *C. reevesii* showed relatively higher reserved VC levels and that renal VC content increased significantly after recovery in Chinese soft-shelled turtle, perhaps because of increased production of VC, as the kidney is the tissue for VC production in turtles. However, plasma VC levels of the three turtles showed consistent changes, and all increased significantly during the anoxia period. After recovery, plasma VC content of the three-keeled pond turtle and the snapping turtle went down to the control level, while that of the soft-shelled turtle remained at a high level (Figure 11). These results may indicate that although both *C. reevesii* and *P. sinensis* rely on VC in antioxidant defense, their strategy is different. The three-keeled pond turtle kept a higher basal VC reserve, and its renal VC synthesis ability did not fluctuate much under anoxia stress, while the soft-shelled turtle maintained a lower VC reserve, but its VC synthesis could be induced in the kidney in order to cope with oxidative damage after oxygen supply was restored. In contrast, the superior anoxia tolerance of the snapping turtle may be mainly due to high-component antioxidant enzymes rather than small-molecule antioxidants. In addition, unlike *C. reevesii* and *C. serpentina*, the changes of CAT mRNA expression, GPx activity and GSH content in the brain of *P. sinensis* showed preparation for an oxidative stress (POS) strategy.

## 5. Conclusions

The antioxidant defense system showed different changing patterns in response to anoxia stress of the three freshwater turtle species. The three-keeled pond turtles and the snapping turtles tended to maintain homeostasis of the antioxidant system. Their storage antioxidants were enough to protect the body from oxidative damage. The brain of the snapping turtles had high structural antioxidant enzyme activity to keep a consistently high antioxidant defense level. However, the Chinese soft-shelled turtle tended to adjust the antioxidant defense system induced by stress during the period of anoxia so as to cope with the damage caused by anoxia quickly and cope with the oxidative stress that may occur after recovery.

## Figures and Tables

**Table 1:** Gene name, GeneBank accession number, forward and reverse primer sequences and primer amplicon size for target genes analyzed.

Gene Name	Accession No.	Forward Primer	Reverse Primer	Amplicon Size (bp)
GAPDH	NM_001286927.1	TTCATGGCACTGTCAAGGCT	GGTTGACGCCCATCACAAAC	243
Cu/Zn SOD	JX470524	TGCAGGTGCTCACTTCAATCC	CAACATGCCTCTCTTGATCTTGTG	68
Mn SOD	JX470525	GCCATCAAGCGTGATTTCG	CTGATACTGCTGTCAGCTTCTCCTT	61
CAT	JX452102	GCAGCGCTTCAATAGTGCAA	GTTCATCTTCTTTCAGCACTTTGG	80
GPx1	KC357250	GGAGCCCTTCAAACGCTACAG	TGAGGAGGCGCTGGATGT	71
GPx3	JX470527	AACCAGTTTGGCAAGCAAGAG	CGGGCCGGACGTATTTC	70
GPx4	JX470528	AGTAAGATAGAGGTCAACGGGAACA	TTCCTTTGGGCTGATCTTTCA	70

## Data Availability

All datasets generated during this study are included in this published article, and all materials generated during this study are available upon request.
